# Winter Ecology of the Hen Harrier, *Circus cyaneus*: Bridging Behavioral Insights and Conservation Requirements

**DOI:** 10.3390/ani15071057

**Published:** 2025-04-05

**Authors:** Remo Probst, Renate Probst

**Affiliations:** Ornis—Biology Engineering Office and Research Institute, Dr. G. H. Neckheimstr. 18/3, A-9560 Feldkirchen, Austria; renate.probst@ornis-institut.at

**Keywords:** hen harrier, *Circus cyaneus*, winter ecology, conservation behavior, land use change, behavior-based management, behavioral indicators, territoriality

## Abstract

The hen harrier, *Circus cyaneus*, is a migratory raptor that relies on open grasslands as key winter habitats in Central Europe, where it predominantly preys on common voles, *Microtus arvalis*. However, habitat loss due to agricultural intensification, land use change, and human disturbances increasingly threatens these landscapes. Using the framework of conservation behavior, this study examines how hen harriers respond to human-induced environmental changes, how behavior-based habitat management can support conservation, and how behavioral indicators such as territoriality inform conservation planning. Based on 278 h of field observations, we analyzed space use, foraging ecology, time and energy budgets, and territoriality of wintering hen harriers. Our results indicate that non-territorial females require ~20% more energy intake than territorial females, reflecting the advantage of defending vole-rich habitats. This corresponds to an estimated daily intake of up to 8 voles for non-territorial females, compared to 4–6 voles for territorial ones. Winter territory establishment was primarily driven by vole availability, and habitat degradation due to grassland loss impaired territory formation, influencing energy allocation and conservation prospects. Our findings highlight the role of territory establishment as a quantifiable behavioral indicator for habitat quality and conservation planning. At a landscape scale, habitat alteration and loss, anthropogenic disturbances and top-down effects further shape hen harrier distribution, emphasizing the need to account for these factors in conservation strategies. Finally, we identify open research questions, especially regarding large-scale environmental pressures and cross-seasonal effects.

## Simple Summary

The hen harrier is a migratory bird of prey that depends on open grasslands as key winter habitats in Central Europe, where it hunts small mammals, particularly common voles. However, these landscapes are increasingly threatened by agricultural intensification, human disturbance, and soil sealing, which reduce prey availability and limit suitable foraging areas. Through direct field observations, we studied how males, territorial females, and non-territorial females use different strategies to cope with winter conditions. Territorial females defend high-quality habitats and require less energy than non-territorial females due to prolonged periods of sitting without frequent foraging flights, highlighting the advantage of stable access to prey. However, habitat degradation forces behavioral trade-offs, which could impact the survival and conservation of wintering hen harriers. Our findings show how behavioral insights can help improve conservation strategies by preserving prey-rich grasslands and ensuring suitable conditions for territory establishment. Protecting open landscapes is essential not only for hen harriers but also for other grassland-dependent raptors in human-altered environments.

## 1. Introduction

Raptors, as apex predators and ecological sentinels, are critical indicators of ecosystem integrity due to their position at the top of the food web [1,2]. Key fields of study for understanding their ecological roles and conservation include population and ecosystem dynamics, community interactions, trophic relationships, conservation biology, and behavioral ecology [3,4]. Conservation behavior provides a valuable framework for bridging animal behavior with conservation challenges [5]. This scientific approach explores how behavioral aspects, ranging from movement and space use, foraging and predator-prey interactions, to social systems and reproduction, interact with human-induced environmental changes. By addressing three core domains, namely anthropogenic impacts on animal behavior, behavior-based management as conservation strategies derived from behavioral insights, and behavioral indicators as quantifiable measures of processes relevant to conservation, it enables both the analysis of complex conservation requirements and the development of targeted management strategies [6,7].

Members of the genus *Circus* have evolved to thrive in open grasslands and wetlands throughout their breeding, migratory, and overwintering ranges. Known for their buoyant, ground-skimming quartering flight, long-distance foraging supported by low wing loading, terrestrial nesting, and aerial prey transfer [8,9], they exhibit diverse distributional, morphological, ecological, and behavioral traits. The hen harrier, in particular, stands out due to its extensive Eurasian distribution, almost ‘owl-like’ hearing abilities, and frequent polygynous mating, even forming loose colonies [10]. As partial migrants, it vacates breeding areas in northeastern Europe and northern Asia, reaching wintering sites, including our Central European study regions, via broad-front migration [11]. The migration and wintering period represents a particularly vulnerable phase for raptors like the hen harrier, during which pressures such as reduced prey availability, adverse weather conditions, and human-induced risks significantly affect their fitness and survival [12,13,14]. In the case of hen harriers, grasslands play a pivotal role as winter foraging habitats by supporting vole populations that constitute a key prey resource [15]. Interestingly, the larger females, exhibiting reversed sexual dimorphism, establish and vigorously defend territories during the non-breeding season in high-quality habitats [16]. The hen harrier provides a particularly valuable model species for studying the behavioral adaptations of raptors in winter, as its reliance on open grassland habitats and the distinction between territorial and non-territorial individuals offer important insights into the ecological needs and conservation challenges faced by wintering raptors in agricultural landscapes.

For the hen harrier, expert-driven assessments of endangerment drivers, conservation measures, and knowledge gaps have primarily focused on the breeding season [17,18]. Our study on overwintering harriers addresses key questions within the three domains of conservation behavior, examining both how hen harriers adapt their behavior to human-induced environmental changes and the implications of these adaptations for their conservation requirements. Regarding anthropogenic impacts [16,19,20,21,22,23,24,25,26,27], we examine how human-modified environments influence key behavioral aspects of overwintering hen harriers, including prey selection, habitat use, time and energy budgets, and the establishment of temporary winter territories, with particular attention to sex differences in shaping these behaviors and their relation to nutritional needs. In the context of behavior-based management [19,20,21,22,23,24,25,26], we explore how behavioral patterns can be used to guide the development of habitat-specific strategies to improve prey availability and territorial formation for overwintering hen harriers. We hypothesize that habitat features such as grassland availability and vole abundance, ensuring territory formation, are critical for designing conservation strategies guided by behavioral evidence. Behavioral indicators [16,22,27,28,29,30], such as space use, foraging behavior, and time budgets, provide direct proxies for habitat quality and environmental pressures, and we will demonstrate that the establishment of territories serves as a particularly robust measure of both. By linking behavioral insights, such as habitat use and territorial behavior, with conservation strategies, our findings will mitigate human-induced pressures and guide management decisions aimed at enhancing winter habitats for hen harriers in agroecosystems.

In the discussion, we will additionally address broader topics at the landscape scale, including habitat use, prey availability, top-down effects, competitive interactions, and anthropogenic threats apart from agricultural intensification. We will conclude by emphasizing the need for enhanced international collaboration to ensure the long-term conservation of hen harrier populations [31] and highlighting several open questions for future investigation. Addressing these questions will not only enhance practical conservation efforts but also contribute to the scientific advancement of conservation behavior in raptors.

## 2. Materials and Methods

### 2.1. Taxonomy and Identification

We followed the taxonomy and nomenclature of the IOC (International Ornithological Congress) World Bird List, version 14.2 (updated 17 August 2024, by F. Gill, D. Donsker, and P. Rasmussen: https://www.worldbirdnames.org/new/bow/raptors/ (last accessed on 21 January 2025)). According to this classification, the Eurasian hen harrier, *Circus cyaneus*, is distinguished at the species level from the Nearctic northern harrier, *C. hudsonius*. Other so-called ‘ring-tail’ harrier species, the pallid harrier, *C. macrourus*, and the Montagu’s harrier, *C. pygargus*, occur in Central Europe during winter months only as vagrants and can be identified with appropriate experience and reliable identification literature [32,33,34].

When sexing and aging hen harriers, we adhered to the criteria outlined in [35]. Key defining characteristics encompassed aspects of ‘jizz,’ including shape, size, and powered flight style, as well as diagnostic features such as eye color, plumage pattern, and coloration. Young birds in their first winter of life, still bearing their first generation of remiges, are per definition juveniles [32], all older birds were classified as adults.

The possibility of individual (within-season) recognition of female-type hen harriers and northern harriers on the basis of distinctive plumage characteristics and sometimes also molting limits/contrasts or broken feathers has been conclusively demonstrated by other authors [28,29,36]. Tricky identifications were supported with high-quality images for confirmation. However, adult male hen harriers are typically not identifiable at the individual level [33]. For instances where two or more adult males were observed simultaneously or in the presence of distinctly recognizable individuals, separate protocols were applied. In all other cases, observations were attributed to a new individual if no adult male had been observed in the same study area for two consecutive days of monitoring. The total number of hen harrier individuals was determined by summing annual counts across years.

### 2.2. Study Areas

**Figure 1:**
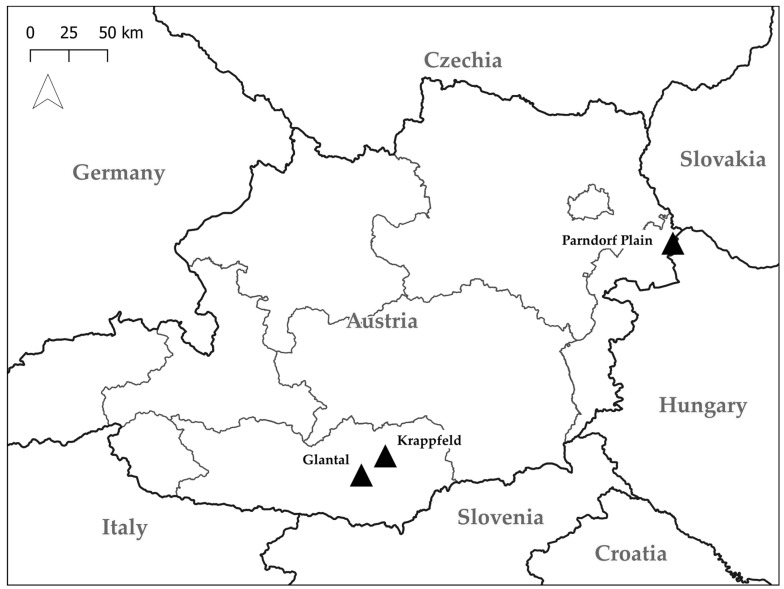
Locations of the main study areas (black triangles) in the Austrian provinces of Carinthia (Glantal and Krappfeld) and Burgenland (Parndorf Plain).

Field surveys for hen harriers were conducted in the Austrian provinces of Carinthia and Burgenland (Figure 1), covering the winters from 2020/21 to 2024/25. In the primary study region of Carinthia, the surveys focused on the inner-alpine Klagenfurt Basin, specifically the Krappfeld (centered at 46.832° N, 14.471° E; 600 m a.s.l.) and the Glantal (centered at 46.729° N, 14.259° E; 500 m a.s.l.). Additional observations were carried out near Sankt Jakob (46.564° N, 14.049° E; 470 m a.s.l.) in the southern Klagenfurt Basin. The mean January temperature for 2020–2024 at the Klagenfurt meteorological station was −1.24 ± 1.42 °C (https://www.zamg.ac.at/cms/de/klima/klima-aktuell (last accessed on 21 January 2025)). The habitat is predominantly intensively farmed agricultural land with varying proportions of grassland and winter catch crops, such as rapeseed. Within the Krappfeld (~7 km^2^) and Glantal (~3.5 km^2^), structural elements—such as individual trees, hedges, or forest fragments—are sparse, and there are only peripheral settlements. Both study areas can be described as ‘flat agrarian islands’ surrounded by extensive woodlands and mountains.

In Burgenland, the surveys were conducted in the northern part of the province, specifically on the Parndorf Plain near the village of Nickelsdorf (47.938° N, 17.069° E; 130 m a.s.l.). The prevailing Pannonian climate is characterized by relatively cold winters despite the low altitude, with a mean January temperature of 2.62 ± 1.11 °C recorded in Eisenstadt from 2020–2024 (https://www.zamg.ac.at/cms/de/klima/klima-aktuell (last accessed on 21 January 2025)). This habitat is an open, shallow ‘agricultural steppe’, forming part of the Little Hungarian Plain (~8000 km^2^). Within this expansive area, hen harriers typically occupy meadow remnants, catch crops and set-aside sites that are rich in vole prey [37].

In all our study areas, hen harriers are exclusively passage migrants and winter visitors. The nearest regularly breeding population, located in the Austrian Waldviertel (37–38 pairs in the highest recorded year 2024 [38]) and neighboring regions of the Czech Republic [39,40], occurs approximately 150 km northwest of the Parndorf Plain. This breeding population is isolated within Central Europe from the northern core range [41].

### 2.3. Prey Identification and Consumption

We identified prey (and corresponding capture habitat, Section 2.5) through direct observation. This method was chosen instead of pellet analysis from roosting sites, as the latter would be detached from the behavioral protocol. The dry, open-land habitats of our study areas are primarily inhabited by the common vole, *Microtus arvalis*, which is morphologically distinguishable even at a distance by its compact body shape, short ears, and short tail. Prey captured in tall vegetation, such as rapeseed fields, is often transported to open areas by hen harriers (presumably as an anti-predator strategy), facilitating genus identification. Other small mammals, such as moisture-dependent vole taxa like the field vole, *Microtus agrestis*, and the significantly larger water vole, *Arvicola terrestris*, are essentially absent [42,43,44]. Further cathemeral or polyphasic species such as the bank vole, *Clethrionomys glareolus*, and the common shrew, *Sorex araneus*, are very rare in sparsely vegetated habitats [45,46,47], whereas members of the long-tailed genus *Apodemus* are primarily nocturnal [48,49,50,51]. Although other small mammals, including Muridae, Soricidae, and Cricetidae (subfamily Arvicolinae), cannot be ruled out as prey, the near-exclusive observation of short-tailed *Microtus* in handled catches and their dominance in the studied habitat support the assumption that common voles were the main prey.

Energy allocation for individual hen harriers was based on the actual consumption of prey. In cases of successful food parasitism [52], the energy content of the prey was assigned to the pirating hen harrier that actually consumed it. Conversely, when a hen harrier fell victim of theft, it forfeited the energy value of the prey it had originally captured.

### 2.4. Time and Energy Budgets

We monitored hen harriers during the primary wintering period, from November to January [11], to minimize the influence of transient migrants present only briefly. Our survey methodology followed that described in [22,28,29], originally developed to study the northern harrier, the ecologically vicariant species of the Nearctic. Harriers were primarily observed from a vehicle by two experienced observers using 10 × 42 binoculars or 20–60× spotting scopes. This minimally invasive approach was made feasible by the open landscape with low visual barriers and the site fidelity exhibited by territorial or stationary non-territorial hen harriers. In complex scenarios, one observer watched the harrier(s) while the second maintained the behavioral log.

First, the sex, age, and identity of each bird were determined (Section 2.1). Territorial females were recognized by their distinct defensive behaviors, including chasing and escorting non-territorial hen harriers and other raptors, territorial calls, and the adoption of the aggressive ‘Kong position’ on the ground (for details, see [16,28,30]). Since the food-rich female territories also represent highly attractive foraging grounds for other harriers and vole-hunting raptors, daily conflicts initiated by the dominant territorial females occur. In our dataset, these involved on average one displacement per hour and often included prolonged guarding behavior lasting several minutes. Especially other female hen harriers are not tolerated and are fiercely attacked. The individuals identified as territorial consistently emerge as winners in these encounters, successfully repelling all conspecific intruders. Although a few males defended their core home range against other males and even attacked the distinctively larger females, we classified all males as non-territorial (sensu [16]). The following three groups of hen harriers were distinguished for our analyses: (1) males, (2) non-territorial females, and (3) territorial females. Data collected during the rare periods of continuous snow cover > 5 cm were excluded from this categorization due to limited sample size, as most individuals quickly departed under these adverse conditions.

The recording protocols were maintained continuously, with observed behaviors assigned to 30-sec intervals. To facilitate the conversion of time budgets into energy budgets, hen harrier behaviors were grouped into five overarching categories, following the approach of [22]: (1) sitting (including eating and calling from a perch), (2) soaring, (3) quartering flights (in search of voles), (4) non-aggressive flying (without foraging), and (5) high-speed flying such as territorial chasing, mobbing, bird hunts, and escape flights from predators.

The calorie was used as the unit of measurement for energy. The basal metabolic rate (BMR) for nonpasserines, including hen harriers, was calculated following the equations provided in [53], with separate calculations for resting during the day and resting at night (Equations (1) and (2)). The body mass of hen harriers required for these calculations was obtained from [15]. Wintering birds from neighboring Italy and Hungary had an average weight of 507 g for females and 350 g for males.

log Resting Daytime = log0.0247 + 0.729 ∗ log(mass in g),(1)

log Resting Nighttime = log0.0193 + 0.734 ∗ log(mass in g)(2)

The BMR of female hen harriers is 2.317 kcal/h during the day and 1.854 kcal/h at night, while for males, it is 1.770 kcal/h during the day and 1.422 kcal/h at night. In accordance with [22] and the original literature cited therein [54,55,56,57], multipliers were applied to estimate the energy costs for the five behavioral categories of hen harriers described above. The resulting values and corresponding multipliers are presented in Table 1. Notably, ref. [27] suggests a multiplier of approximately 10 for quartering flights, differing from the value of 8 used in [22], resulting in energy costs of 23.170 kcal/h for females and 17.700 kcal/h for males. This alternative factor was incorporated as a variation in the energy budget calculations (Section 3.3). The winter night was standardized to 14 h and the daylight to 10 h following the methodology in [22]. This approach ensures consistency and comparability when calculating daily energy budgets and nutritional requirements.

We used a caloric value of 1.301 kcal per gram of biomass for wintering common voles, based on calorimeter measurements from [58]. The body mass of (captured) common voles varies significantly depending on factors such as age, sex, season, population cycle, and predator selection (e.g., [59,60,61,62,63,64]). To account for this variation, we conducted calculations using a low average weight of 17 g [27] and a high average weight of 25 g [60,61]. These values correspond to an energy content per vole of 22.1 kcal and 32.5 kcal, respectively. The lower value was also applied to the few small birds that were captured (cf. [65]).

### 2.5. Vole Abundance Index and Territoriality

We quantified common vole availability using the surface index method described by [66], which exhibits a strong correlation with actual vole abundance [67]. This method involved conducting line transect surveys and recording the presence or absence of vole droppings at 10-pace intervals. The resulting abundance index, expressed as the percentage of intervals containing droppings, serves as an indicator of vole availability. Surveys were carried out within 11 plots located in the core study areas, with plot sizes ranging from 30 to 50 ha. The plots were delineated based on habitat suitability and the hen harrier’s ability to defend territories ranging from ~30–100 ha [29,36]. Each plot included at least one established hen harrier territory during the study period and was monitored for one to five years, depending on the site. The parcels were selected for their active use by hen harriers as hunting grounds, focusing on agricultural grasslands characterized by low or sparse vegetation. Vegetation types included permanent low-growing or autumn-mown forage crops, such as meadows, pastures, and clover-grass mixtures, along with sparse catch crops like fast-growing rapeseed mixes. Each utilized parcel was surveyed once, typically along its full extent. The vole index per ha was calculated based on parcel surveys and the proportion of vole-free or unsuitable areas within each study plot.

To assess the influence of vole availability on hen harrier territory establishment, we recorded the presence or absence of a territorial harrier for each surveyed plot and winter within one month of the vole count. Most plots hosted either one or no territorial harriers per winter. However, in some cases, vole indices were measured multiple times within the same winter. This occurred either after a significant loss of foraging areas, mainly due to meadow plowing, which led to territory abandonment (*n* = 1), or when a non-territorial harrier established a territory weeks after the initial observation following the departure of competing individuals (*n* = 1). In one case, where multiple territorial harriers occupied a single plot (30 ha with four territorial females), the vole index was calculated proportionally for the areas each individual used. In total, 26 plot-years were available to test the relationship between territoriality and vole abundance.

### 2.6. Land-Use Change

We analyzed the short-term trend of forage crops in our inner-Alpine study region, where suitable habitats are limited due to extensive forest cover and mountainous terrain. Forage crops are particularly valuable because, unlike catch crops, they are expected to be perennial, offering stable habitats that facilitate the establishment and cycling of vole populations. A subset of four study plots (Section 2.5) was used, with the proportional area of forage crops recorded annually each autumn using GIS-based area calculations. Monitoring commenced in different baseline years (see Section 3.4).

### 2.7. Data Analysis

For this analysis, 278.2 h (16,692 min) of observations were conducted, covering > 100 individual hen harriers from southern and eastern Austria. Of these, 222.2 h (13,329 min) exclude observations during periods of continuous snow cover > 5 cm. The latter dataset is distributed among the three predefined hen harrier groups (with an adult-to-juvenile ratio in parentheses) as follows: 76.6 h for males (*n* = 52, 48:52), 78.1 h for non-territorial females (*n* = 40, 45:55), and 67.5 h for territorial females (*n* = 16, 62.5:37.5).

To compare two non-normally distributed variables, Mann-Whitney U tests were conducted [68]. For comparisons involving more than two groups, Kruskal-Wallis tests were applied, followed by Dunn-Bonferroni post-hoc tests to identify specific group differences [69]. A logistic regression analysis was performed to assess the dichotomous variable territoriality, including two predictor variables [70].

## 3. Results

### 3.1. Energy Intake: Diet, Consumption Rate, and Capture Sites

Hen harriers wintering in southern and eastern Austria were identified as highly specialized predators of microtines. Among the 274 documented prey items captured or kleptoparasitized, 98.2% consisted of small mammals (*n* = 269), with direct observations revealing an almost exclusive reliance on common voles, *Microtus arvalis*. Other mammalian species were suspected only in a few isolated cases, and in one instance, we confirmed the capture of a shrew, *Sorex*/*Crocidura* spp. Avian prey accounted for only 5 individuals, including 2 common linnets, *Linaria cannabina*, 1 common reed bunting, *Emberiza schoeniclus*, 1 Eurasian chaffinch, *Fringilla coelebs*, and 1 unidentified small bird captured at a distance.

The consumption rate of successfully captured or kleptoparasitized prey was 95.3%. Food parasitism of hen harriers occurred intraspecifically (*n* = 8) and on common kestrels, *Falco tinnunculus* (*n* = 2). Interspecifically, prey was lost to the red kite, *Milvus milvus* (*n* = 1), the common buzzard, *Buteo buteo* (*n* = 3), and the Saker falcon, *Falco cherrug* (*n* = 1).

Voles were captured predominantly in forage crops (69.9%), followed by catch crops (22.0%), and only exceptionally in pastures (0.8%); however, the latter covered only a negligible fraction of our study regions. The proportion of other habitat types used as capture sites, including bare arable fields, winter cereals, and stubble fields, which together accounted for a considerable share of the study area (Section 3.4), was low (7.3%).

### 3.2. Time Budget and Energy Expenditure: Sex and Territoriality Effects

**Figure 2:**
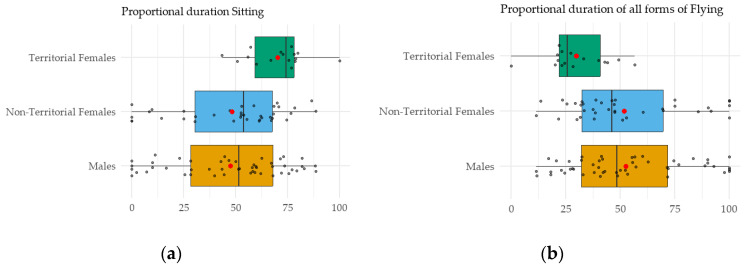
Proportional duration of the behavioral category sitting and the pooled flying categories during daylight hours: (**a**) Territorial females allocated a significantly greater proportion of time to sitting and (**b**) exhibited reduced flight activity compared to males and non-territorial females, despite the necessity of territory defense against intruders. This underscores the effect of vole-rich territories, where reduced hunting requirements allow for a shift in time budgets. The box indicates the standard deviation, the vertical line represents the median, and the red dot marks the mean.

Aggregating the time allocation of diurnal behaviors across all individuals (including only observation min from periods with continuous snow cover < 5 cm), Table 2 presents the time budget of the three hen harrier groups. Since individual hen harriers were monitored for varying observation times, behavioral categories had to be analyzed as relative percentages rather than absolute durations. For each individual, we summed the total min spent in a given behavior and subsequently calculated its relative contribution. The proportion of time spent sitting (Figure 2a) differed significantly among the three groups (H = 11, df = 2, *p* = 0.005), with territorial females sitting significantly longer than both males (*p* = 0.006) and non-territorial females (*p* = 0.011). There was no difference between males and non-territorial females (*p* = 1.000). No significant differences were detected at the group level for the other four behavioral categories. However, when pooling all flight-related behaviors (Figure 2b), a significant group-level difference was found (H = 11, df = 2, *p* = 0.005), with territorial females exhibiting significantly lower flight activity than both males (*p* = 0.006) and non-territorial females (*p* = 0.011). Males did not differ from non-territorial females (*p* = 1.000).

The total energy expenditure, derived from the energy costs presented in Table 1 and the time budget in Table 2, is summarized in Table 3. In line with the time budget, the proportional contribution of the behavioral category sitting differs significantly among the three harrier groups (H = 11, df = 2, *p* = 0.004). Due to their prolonged sitting periods, territorial females exhibit significantly higher energy expenditure compared to both males (*p* = 0.004) and non-territorial females (*p* = 0.010), while no difference is observed between males and non-territorial females (*p* = 1.000). When pooling the energy expenditures across the four flight categories, a significant group-level difference was detected (H = 11, df = 2, *p* = 0.004), with territorial females investing proportionally less energy in flight than males (*p* = 0.004) and non-territorial females (*p* = 0.010). In contrast, the latter two showed similar levels (*p* = 1.000).

Consequently, territorial females’ total diurnal energy expenditure approximates that of males, despite the latter exhibiting a considerably lower BMR and greater foraging agility. In contrast, non-territorial females display the highest diurnal energy expenditure among the three groups. Considering the full 24-h cycle including nighttime resting, territorial females maintain an intermediate total energy expenditure between males and non-territorial females. Thus, despite the energetic demands associated with territorial defense, the overall energy balance clearly favors territorial behavior in female hen harriers.

### 3.3. Energy Budget: Food Requirements and Foraging Efficiency

The daily food requirements to maintain energy balance in overwintering hen harriers can be derived from Table 4, which accounts for the differing energy content of voles and the variability in energy expenditure associated with quartering. On average, males and territorial females require a similar amount of food per day (~3.5–5.5 and ~4–6 voles, respectively), while non-territorial females need substantially more (~5–8 voles/day). The latter have a higher BMR than males and expend significantly more energy than territorial females due to the greater costs of vole hunting and generally increased flight activity.

Under the scenario with the highest energetic demand, males must consume one vole (or an energetically equivalent prey item) every 0.5 h, territorial females every 0.6 h, and non-territorial females every 0.8 h. All three hen harrier groups met these requirements, with territorial females consuming 0.9 voles/h, males 1.0 voles/h, and non-territorial females 1.1 voles/h. However, when considering only active quartering times, territorial females required, on average, only 15.5 min of foraging/vole, while males needed 16.1 min and non-territorial females 19.1 min. This highlights the advantage of prey-rich territories and refutes the potential methodological bias that territorial birds appear to have lower energy expenditure in direct observations simply because their prolonged sitting behavior is more easily detected (cf. [71]).

During periods with snow depths of 7–24 cm, the consumption rate averaged 0.7 voles per hour across all three hen harrier groups. Considering methodological uncertainties, this appears to be at the threshold for maintaining energy balance. Indeed, under conditions of persistently high snow levels, the study areas were abandoned by the hen harriers.

### 3.4. Vole Availability and Territoriality

**Figure 3:**
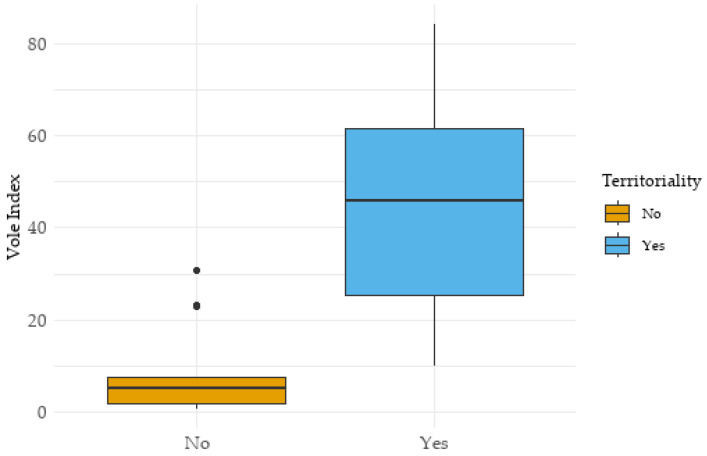
The establishment of hen harrier territories in Central Europe was strongly driven by vole availability, measured as an abundance index per ha. The difference between areas with and without territorial hen harriers was statistically significant.

A highly significant difference in the vole abundance index between territorial and non-territorial individuals was detected, with a large effect size (U = −3.86, *p* < 0.001, r = 0.757; Figure 3). For territorial females (*n* = 12), the index averaged 45.2 ± 25.0, whereas during periods without established territories on the study plots (*n* = 14), it remained low at 8.5 ± 9.8. A logistic regression revealed that within the study plots, vole availability (odds ratio = 1.149, *p* = 0.02) was a stronger predictor of territoriality than the available area of forage and catch crops suitable for hunting by hen harriers (odds ratio = 0.996, *p* = 0.888). An inspection of variance inflation factors (VIF) indicated no problematic multicollinearity between the predictors (vole availability and huntable area), with all VIF values < 5. Hen harrier territories were established only when a specific threshold of vole density was exceeded, as high prey availability reduced time constraints for foraging and also allowed for effective territory defense, particularly against intraspecific competitors.

### 3.5. Habitat Alteration

**Figure 4:**
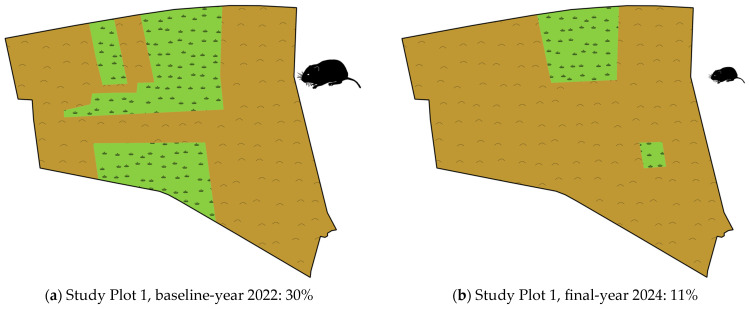

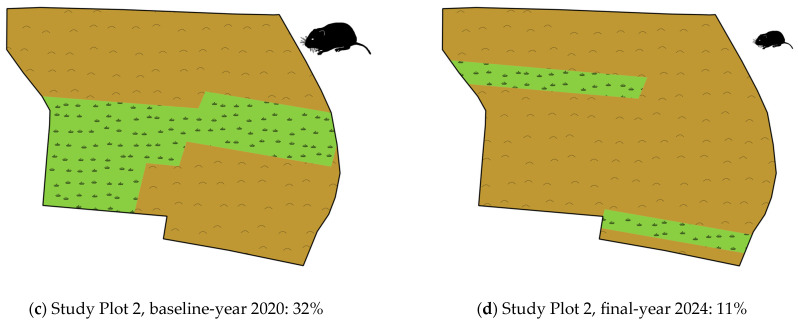
Change in forage crop proportion (%) on two inner-alpine study plots. Within just three and five years, respectively, the huntable areas for hen harriers (shown in green) on both study plots declined by approximately two-thirds. In the early years, hen harrier territories were established; however, in the final year, only non-territorial individuals remained, correlating with the low proportion of meadows.

Prey availability is determined by vole density and the extent of small mammal habitats accessible to hen harriers. While vole density is primarily governed by intrinsic population cycles, habitat extent is predominantly shaped by anthropogenic agricultural land management. In the four main inner-alpine Carinthian study plots, the land cover balance of forage crops exhibited a pronounced decline. During the baseline years (2020: *n* = 1; 2022: *n* = 2; 2023: *n* = 1), forage crop proportions averaged 33.0% ± 14.9 (median: 31), but by the winter of 2024/25, they had markedly decreased to 15.5% ± 15.6 (median: 11). Although the decline in forage crop proportions within the study plots was apparent, statistical analysis was not conducted due to the limited number of sampled sites. Figure 4 illustrates this trend using two representative study plots. In these cases, the vole availability index was consistently moderate to high in both the initial and final years. Hen harrier territories were established only when foraging areas were spatially extensive, but none formed in the final winter, as forage crop proportions declined below critically low thresholds.

## 4. Discussion

Our study is grounded in the well-established framework of conservation behavior, characterized by its logical, hierarchical, and parsimonious structure [6]. Wintering hen harriers serve as a model species to address the three core domains of conservation behavior: (I) understanding behavioral responses to human-induced environmental changes, (II) behavior-based ecosystem management, and (III) the use of behavioral indicators in conservation. Beyond our immediate findings, we discuss (IV) broader ecological and conservation aspects of wintering hen harriers at the landscape scale and conclude with (V) key questions for further research.

(I) Anthropogenic impacts on the behavioral responses of wintering hen harriers: Regarding space use as a key behavioral parameter, wintering hen harriers in Central Europe largely depend on human-maintained or cultivated grasslands for hunting, daytime resting, and partly for nighttime roosting. This raptor is a specialized predator of rodents and small birds [15]. While certain northern regions show a prevalence of avian prey [72], voles dominate the hen harrier’s winter diet in mainland Europe [72,73,74,75,76,77,78]. For instance, common voles made up ~99% of winter prey in southwestern Slovakia, adjacent to one of our study areas [79]. Likewise, our direct observations [80] in southern and eastern Austria underscore the importance of the common vole as a keystone species [81], while other small mammals and birds contributed only marginally to recorded prey items. Rare attempts on large prey, such as the grey partridge, *Perdix perdix*, and the mistle thrush, *Turdus viscivorus*, reflect opportunistic behavior [82] but were never successful in our observations. Wintering hen harriers are thus typical small mammal foragers with low dietary richness, where diet entropy peaks only when aggregated across habitats and study areas [83]. Common voles are mainly captured in forage crops, while small birds, including common linnets and reed buntings, often overwinter in catch crops such as mixed rape cultures. Unlike natural habitats such as moorland, steppe or tundra, Central Europe’s human-shaped winter landscapes provide secondary habitats that mimic structural conditions of natural open-land ecosystems and support the hen harrier’s specialized foraging behavior. Similar to its main prey, the common vole, which relies on moderately dry grasslands [84], the hen harrier thrives in these secondary habitats. Additionally, many roosting sites are located in catch crops, though hen harriers often spend the night in reed beds [37] and occasionally in trees [85].

We developed time and energy budgets based on direct field observations. Since the surveys were consistently conducted by the same two researchers, observer bias was minimized [86]. Data were collected for our research questions on three predefined groups of hen harriers: territorial and non-territorial females, as well as males. To our knowledge, males have not yet been explicitly considered in comparable analyses. Using five defined behavioral categories, territorial females allocated significantly more time to sitting, while non-territorial females and males dedicated more time to hunting and flying. These differences in time allocation directly affect their diurnal energy budgets. We acknowledge uncertainties in absolute estimates due to unaccounted thermoregulation costs [87], metabolizable energy proportions [88], and the use of general rather than raptor-specific BMR predictions [89]. However, for reasons of direct comparability, we followed the approach of [22]. We assume that relative differences in energy expenditure between behavioral classes remain consistent regardless of the specific estimation method. Territorial females expend significantly less energy than non-territorial females and exhibit energy expenditure patterns closer to those of males. Notably, female hen harriers are disadvantaged in hunting compared to males, as they are less agile and, unlike adult males, have darker plumage, making them more conspicuous to small mammal prey [90,91,92]. Non-territorial females demonstrated a maximum daily energy expenditure of approximately 169 kcal, comparable to that of the slightly larger northern harrier (161 kcal/day, [22]). In contrast, territorial hen harriers had a significantly lower maximum requirement of 135 kcal/day. These findings highlight the adaptive value of territoriality under human-induced environmental pressures, where resource-rich areas allow individuals to minimize energetically costly activities.

By varying nutritional requirements based on vole energy content and hunting-related energy expenditure, we estimated that wintering hen harriers require 3.5–8.0 voles/day to maintain a balanced energy budget. This aligns roughly with estimates from [22,27], who suggested a daily requirement of 4 house mice, *Mus musculus*, or 10.2 voles. While the necessary success rate is achieved across all three hen harrier groups, non-territorial females, with their higher energy expenditure, must capture up to two additional voles/day (~20%) compared to territorial females. Territorial females benefit from resource-rich human-altered habitats that reduce the need for energetically costly hunting, despite the efforts required for territorial defense. Males, with their lower BMR, require less food overall. Limited data from periods of closed snow cover indicate a significant decline in hunting success, emphasizing the need for studies on behavioral adaptations under these conditions.

(II) Behavior-based conservation strategies for wintering hen harriers: In Europe, grasslands critical for the hen harrier and many other farmland birds have declined significantly in both extent and functionality [93,94]. This decline is driven by habitat conversion, urban expansion, insufficient policy interventions, and the loss of extensive grazing systems, resulting in a 47% reduction in permanent grassland (including alpine pastures and mountain meadows) in Austria between 1960 and 2020 [95]. These changes reflect broader biodiversity deterioration, with the European Union reporting a net loss of 190 million grassland birds between 1980 and 2017 [96].

Hen harriers require specific habitat characteristics for foraging and even more specialized conditions for territory establishment. Grasslands and surrogate habitats such as arable meadows, field pastures, and clover sowings should ideally be perennial to support the development of vole populations and their natural cycles. Additionally, they must be low-growing, as prey availability (but not abundance) improves with reduced vegetation height. Studies, including [8], have shown that hen harriers are most successful in hunting small mammals in habitats with low vegetative cover. Similarly, in our study, overwintering hen harriers primarily hunted common voles in forage crops mown in autumn (typically <20 cm, [97]) and in sparsely vegetated or frost-damaged catch crops. Furthermore, connectivity between habitat patches is crucial, enabling energy-efficient movement within and effective defense of territories. While our study found that vole abundance outweighs grassland area for territory establishment, larger territories (>1 km^2^) are generally not occupied, as they cannot be effectively defended. Therefore, conservation efforts should focus on creating and spatially concentrating suitable grassland areas. For example, ref. [29] reported northern harrier territory sizes ranging from ~4–125 ha, while in our study, hen harriers defended daily areas of ~10–100 ha.

Extensive, perennial, and low-growing grasslands fulfill these requirements when properly managed. However, forage crops such as arable grasslands, pasture meadows, and various clover species have declined significantly in Austria, with a 20% reduction in lowland areas (2000–2023), accompanied by increased maize cultivation [95]. Our short-term monitoring confirms substantial habitat losses specifically in forage crops [98] within the inner-alpine hen harrier wintering regions, highlighting the decline of crucial grasslands for the species. This finding underscores the urgent need for habitat protection and rehabilitation. Comprehensive agri-environmental funding for conservation, from grassy margins bordering agricultural fields [99] to expansive grassland areas, can help achieve this goal. Region-specific management strategies tailored to hen harriers are particularly effective in priority areas. For example, in the Netherlands, the introduction of ‘birdfields’—perennial, low-growing, vole-rich areas actively managed and designed to support wintering predators such as the hen harrier and short-eared owl, *Asio flammeus*—has shown promise [100]. Trade-offs and synergies of greening measures with other conservation priorities, including breeding birds, must be carefully assessed during implementation [101,102,103,104]. A recent analysis identified our study regions as biodiversity hotspots for systematic conservation planning [105].

(III): Behavioral indicators of conservation value in wintering hen harriers: Studies on overwintering hen harriers are typically limited to pellet analyses and population trend assessments. In Central Europe, standardized population trends [106] primarily reflect the correlated dynamics of vole populations and their predators, assuming stable habitat availability and anthropogenic disturbances. In contrast, behavioral indicators offer greater predictive power for conservation measures, provided they meet key criteria such as adaptiveness (i.e., the ability to adjust to changing environmental conditions; cf. [107]), measurability, fitness relevance, responsiveness, context dependence, predictiveness, and cost-efficiency [7]. Key behavioral indicators for overwintering hen harriers include space use patterns, foraging success (including bird hunting), and time and energy allocation. These indicators serve as reliable proxies for environmental assessments, demonstrating high adaptiveness by reflecting changes in habitat structure and prey dynamics. However, their practical application depends on the cost-efficiency of data collection methods, making their use contingent on logistical and financial resources.

Hen harriers provide a unique opportunity to study daily-established territoriality during winter, offering significant potential as a behavioral indicator. Winter territoriality is relatively easy to observe and directly linked to individual fitness, as territory holders gain priority access to food resources. It integrates direct factors such as grassland availability and prey density, along with indirect effects like competition, human-induced disturbance, and predation risk [7]. Therefore, territoriality can serve as an assessment tool for behavior-based management. If greening measures fail to establish territories over a vole cycle despite addressing confounding factors (e.g., predator-prey dynamics and anthropogenic disturbances; Section IV), the extent or connectivity of grasslands protected, enhanced, or created through these measures must be considered insufficient. A lack of territory formation affects not only dominant hen harrier females, as non-territorial individuals often adopt a ‘satellite-like’ strategy. These harriers either hunt along the edges of resource-rich territories, briefly intruding for short-term access, or remain inconspicuous by crouching within these territories [16].

(IV) Thus far, we have highlighted the importance of grasslands for wintering hen harriers within preselected ‘optimal’ study sites. Moving beyond this localized habitat perspective, we now adopt a landscape-scale approach to examine the ecological drivers of space use, predation pressure, competitive behavior, and anthropogenic threats beyond agricultural intensification [108,109].

- Habitat use, prey availability, and top-down effects: Hen harriers’ winter habitat use is shaped by the interplay of prey availability and predation risk, particularly in forest-rich regions such as the inner Alps or riparian forest margins. Voles, their primary prey in Central Europe, are most abundant in meadows and fallow land [110], with availability influenced by habitat suitability, vegetation structure, and population cycles [111,112,113]. Although voles also occur in higher Alpine regions [42,114,115], hen harriers almost exclusively use open lowlands with expansive flat terrain for hunting and resting. This preference suggests that top-down effects and reduced predation risk significantly shape habitat selection at the landscape scale [116,117,118], beyond the influence of higher vole densities in lowland areas. We assume that the ‘landscape of fear’ created by potent predators such as the Eurasian goshawk, *Astur gentilis*, strongly influences spatial behavior [119]. Our observations include two unsuccessful goshawk attacks, several instances of hen harriers fleeing when the large predator was hunting pigeons nearby, and repeated circling of harriers above perched goshawks. Among other predators, a domestic cat, *Felis catus*, was the only one observed attempting to capture a harrier. While data on nocturnal threats such as wild boars, *Sus scrofa*, eagle owls, *Bubo bubo*, and red foxes, *Vulpes vulpes*, are scarce [120], these predators may affect roosting site selection [121,122]. Large raptors, including golden eagles, *Aquila chrysaetos*, Eastern imperial eagles, *Aquila heliaca*, and white-tailed eagles, *Haliaeetus albicilla*, overlap with hen harriers in winter habitats, but their specific impact remains unclear. Notably, the latter two species demonstrate significant spatial overlap with overwintering hen harriers and are expanding in Central Europe [123,124].
- Competitive interactions: In our study areas, hen harriers face various competitive pressures during winter, primarily involving resource displacement and kleptoparasitism. Common buzzards and Saker falcons regularly displace hen harriers, forcing them to abandon valuable hunting grounds and prey [125]. Particularly the ‘ubiquitous’ buzzards benefit from structured landscapes, launching their attempts from elevated perches. Large *Falco* species, including Saker, gyr, *F. rusticolus*, and peregrine, *F. peregrinus*, occasionally may injure hen harriers during competitive encounters or, in rare cases, even kill them intentionally [15,126]. Smaller raptors, such as the common kestrel, are regularly robbed of their prey by hen harriers but have also been observed displacing (male) harriers from favorable hunting grounds in isolated cases [127]. The relationship with carrion and hooded crows, *Corvus corone* and *Corvus cornix* (including numerous hybrids in our study areas), is dual in nature: while mobbing crows can disrupt hen harriers’ hunting efforts, they may also serve as an early warning system against predators such as the goshawk, which threaten both species.
- Anthropogenic threats apart from agricultural intensification: Firstly, open landscapes are reduced in size through ecosystem conversion, including permanent soil sealing from expanding settlements or the establishment of new industrial areas [128,129]. Secondly, infrastructure development and recreational activities contribute to ecosystem degradation, fragmentation, and disturbances, thereby reducing habitat quality [130,131,132,133]. Thirdly, rodenticides pose a significant risk to non-target organisms due to their high toxicity, environmental persistence, and bioaccumulative properties. Second-generation anticoagulant rodenticides may negatively affect predators both through reduced vole numbers following rodenticide campaigns and through direct mortality as well as sublethal fitness effects [134,135,136]. The accumulation of rodenticides in bioindicators such as raptors has recently been confirmed in Austria [137]. However, neither the exact extent of these threats, nor their cumulative impact on hen harriers at larger spatial scales, has been quantified.

(V) Open research questions: Although the hen harrier is relatively well studied, numerous questions remain unanswered. Without claiming completeness, we emphasize the importance of international collaboration, interdisciplinary approaches, year-round research, innovative methodologies, and uncovering causal mechanisms [17,18,138,139,140] for effective human–raptor coexistence strategies [141]. (a) Winter habitat requirements, establishment of territories, and ecosystem preservation: Despite widespread recognition of habitat loss and degradation due to urbanization, soil sealing, infrastructure development, increasing anthropogenic disturbances, and declining grassland areas used as hunting grounds, a quantitative understanding of their impact remains limited. The interactive effects of spatiotemporal variation in grassland availability [46,142], vegetation height [143,144], soil fertility [110], and vole population cycles [145,146,147] on winter territory formation in hen harriers require further investigation. The thresholds of this important behavioral indicator, vital for setting conservation measures, should be determined at a regional scale [148,149,150], potentially using an experimental approach. Additionally, it is crucial to assess whether current agri-environmental subsidies are sufficient to effectively conserve wintering habitats for hen harriers. (b) Cross-seasonal carry-over effects: Interdependencies between wintering and breeding areas warrant further investigation [151,152,153], particularly given the species’ complex mating system (e.g., [8,23,154]). Future studies should explicitly consider sex- and age-specific effects. Juvenile hen harriers face high risks during their first dispersal or seasonal migration [12,155], emphasizing the need for safe hunting grounds that function as stepping stones and provide suitable overwintering areas. With increasing experience, immature harriers likely optimize migratory routes [155], benefiting from stable habitat conditions. It can be hypothesized that, for breeding-age individuals, overwintering conditions influence physical condition, territory establishment, mating system dynamics, and reproductive success. It would be particularly intriguing to test whether dominant females with stable winter territories—characterized by high vole availability and suitable habitat conditions—attain superior breeding success or occupy alpha positions in polygynous harems. (c) Large-scale environmental pressures: Overarching negative drivers, such as climate-induced dampening of vole population cycles, which may cause shifts in hen harrier populations at a continental scale, demand further investigation [156]. Incidental rodenticide poisoning and deliberate persecution, an important mortality factor in some breeding areas [157,158,159], also require coordinated international efforts to mitigate their impact. (d) Methodological advances: Future studies should integrate direct observation methods with tracking data, which remain scarce for the hen harrier [85,109,160,161,162]. This combined approach would enhance spatiotemporal resolution, minimize observer bias, and incorporate behavioral parameters, thereby improving ecological interpretations. (e) Nature-based solutions: Interestingly, the hen harrier itself could be integrated into nature-based pest control strategies, similar to established concepts involving the barn owl, *Tyto alba* [163]. Investigating its role as a natural regulator of rodent populations in agricultural landscapes could provide valuable insights for conservation-based land management.

## 5. Conclusions

This study integrates the three core domains of conservation behavior, demonstrating how wintering hen harriers respond to human-induced environmental changes, how behavior-based habitat management can enhance conservation strategies, and how behavioral indicators serve as effective tools for conservation planning.

Our findings confirm that space use, time and energy budgets, and prey selection in hen harriers are shaped by anthropogenic land modifications, with agricultural grasslands functioning as secondary habitats. The species’ reliance on perennial, low-growing forage crops for hunting common voles underscores its vulnerability to habitat loss. Declining grassland or prey availability forces behavioral trade-offs, with territorial females benefiting from lower daily energy requirements than non-territorial females. Conservation efforts should prioritize spatially connected, vole-rich grasslands, as they are critical for territory establishment and energy-efficient foraging. Territoriality functions as a quantifiable behavioral indicator, linking fitness benefits to habitat quality. If habitat restoration efforts fail to enable the establishment of territories, prey abundance and confounding factors, such as disturbance, must be reassessed.

Hen harrier conservation is intrinsically related to behaviorally informed habitat management. Integrating behavioral indicators into conservation planning will refine habitat restoration strategies and strengthen population resilience. Wintering hen harriers act as sentinel species for agroecosystem monitoring [164] and as umbrella species supporting broader conservation targets [165,166], including great bustard, *Otis tarda*, common crane, *Grus grus*, white-tailed eagle, eastern imperial eagle, red kite, rough-legged buzzard, *Buteo lagopus*, Saker falcon, Merlin, *Falco columbarius*, and European golden plover, *Pluvialis apricaria* [167,168].

## Figures and Tables

**Table 1:** Energy costs (kcal/h) of hen harriers for five behavioral categories by sex, with a distinction between daytime and nighttime for sitting. Values were calculated using basal metabolic rate (BMR) multipliers from Equations (1) and (2).

Behavior	Multiplier	Female	Male
Sitting Daytime Sitting Nighttime	1.7	3.939	3.009
	1.7	3.152	2.417
Soaring	7	16.219	12.390
Quartering	8	18.536	14.160
Flying	11.5	26.646	20.355
High-Speed Flying	12.5	28.963	22.125

**Table 2:** Time budget (min, with percentage values in parentheses) across five behavioral categories of hen harriers wintering in Central Europe, classified into three groups based on sex and territoriality.

	**Sitting**	**Soaring**	**Quartering**	**Flying**	**High-Speed Flying**	**Total (Min)**
Males	2871.5 (62.5%)	42.0 (0.9%)	1157.0 (25.2%)	231.5 (5.0%)	289.0 (6.3%)	4591.0
Non-Territorial Females	2696.5 (57.5%)	43.0 (0.9%)	1469.0 (31.3%)	230.0 (4.9%)	249.5 (5.3%)	4688.0
Territorial Females	3016.5 (74.5%)	7.0 (0.2%)	808.0 (20.0%)	87.5 (2.2%)	131.0 (3.2%)	4050.0

**Table 3:** Energy expenditure (kcal) for five behavioral categories of hen harriers wintering in Central Europe. Calculations were conducted for the 10 h of daylight (subtotal) and the entire day, including a fixed value for the 14 h of nighttime resting (total). The harriers were divided into three groups based on sex and territoriality.

	**Sitting**	**Soaring**	**Quartering**	**Flying**	**High-Speed Flying**	**Subtotal**	**Night**	**Total (kcal)**
Males	19.673	0.709	36.899	7.396	9.634	74.312	33.838	108.150
Non-Territorial Females	22.657	1.488	58.083	13.073	15.414	110.715	44.128	154.843
Territorial Females	29.338	0.280	36.980	5.757	9.368	81.724	44.128	125.852

**Table 4:** Food requirements for the three groups of hen harriers overwintering in Central Europe are quantified as the number of vole consumptions required per day to maintain a balanced energy budget. Calculations account for both light (17 g, 22.1 kcal) and heavy (25 g, 32.5 kcal) voles, reflecting their significantly different energy contents. For calculations marked with an asterisk, the quartering costs were adjusted to 10 times the basal metabolic rate (BMR), rather than 8.

	**Requirement (kcal)**	**Voles (22.1 kcal)**	**Voles (32.5 kcal)**	Requirement * (kcal)	Voles * (22.1 kcal)	Voles * (32.5 kcal)
Males	108.150	4.9	3.3	117.448	5.3	3.6
Non-Territorial Females	154.843	7.0	4.8	169.282	7.7	5.2
Territorial Females	125.852	5.7	3.9	135.212	6.1	4.2

## Data Availability

All data, tables, and figures are original. Details on data availability can be obtained from the corresponding author upon reasonable request.
